# Some Thoughts upon Personal Dental Education

**Published:** 1892-01

**Authors:** W. Mitchell

**Affiliations:** London, England


### SOME THOUGHTS UPON PERSONAL DENTAL EDUCATION.[1]

BY W. MITCHELL, D.D.S., LONDON, ENGLAND.

Mr. President and Gentlemen,—I must confess it is with some trepidation that I undertake to write and speak upon a subject that has been discussed and written upon so often, and which has absorbed so much of the attention of the greatest minds of our profession, but still remains as one that must ever lie closest to our conscience; for it is always presenting new phases, as it must necessarily do, being inseparable from a profession that is from its very nature progressive and adaptive; searching here for the cause of phenomena exhibited in as many different ways as there are controlling or modifying influences, or there delving into the mysteries of scientific investigation for the solution of problems that are hidden from us by a coyness that nature alone possesses, and of which she only divests herself after the most persistent importuning on the part of the earnest searcher after truth.

That we are living in an age in which mediocrity has only to be seen to be as readily recognized is a fact that will admit of but little argument, yet there still remains an abundance of it in all professional life, and I think I may safely say, without fear of being refuted, that there is as much to-day in our chosen profession as in any other; deplorable fact that it is, we must face it as it stands, though it is not as we should wish it to be.

Recognizing and appreciating the fact that all effects are the result of causes, either contributory or predisposing, let us look about us, and, if possible, detect the cause for the following effects, —viz., difficulties attending a popular dental education, and lack of stability in the idea of young practitioners.

---

[1] Read before the American Dental Society of Europe, at Heidelberg, August 5, 1891.

The foregoing propositions may be considered separately; but from the very nature of the subject they are so intimately blended that it is impossible to draw a distinct line of demarcation; so intimately do productive causes blend that it is almost forced upon us to go back of the beginning.

There being but two sources of knowledge,—personal and acquired,—it is easy to see that we, as public educators, must be so thoroughly impressed with the responsibility of our position that no operation should be performed, or word uttered, but that carries with it the stamp of reflection, deliberation, and truth; with these ideas as the basis of our creed, there need be but little fear of our occupying the position the most zealous workers of our profession so arduously toil for.

Can we hope for a popular dental education while so many of the profession remain in ignorance? No! certainly not! Let us then break down the barrier that has ever existed to bar the way of progress in every clime and age. But there we are confronted with the question, How are we going to do it? Were it not for the inspiring and friendly light of our societies, whose lamps are kept trimmed and burning by the vigilance and self-sacrifice of those whose life's work has been in the interests of their cherished calling, I must admit the question would be difficult of solution.

To be able to instruct others, we must of necessity possess the knowledge we wish to impart, and also have the ability to demonstrate or didactically convey our ideas of the lesson we wish our pupils or patients to learn.

This brings us to the consideration of our sources of information. We owe it to ourselves, the profession, and the public that a correct system of practice be studied, and a taste cultivated for keeping fully abreast with the times, and a desire to be thoroughly acquainted with all advanced methods, research, and discovery in the closely-allied sciences of dentistry and medicine; being careful to thoroughly scrutinize from different points of observation all alleged facts, and after submitting them to the crucial test of practical application, then, if not found wanting, we can enshrine them in our professional repository and cherish them as gems of the fairest type.

Here, then, is the opportunity of analyzing our own capacity for the performance of a public duty which at some time or another falls to the lot of us all,—*i.e.*, the selection and education of our pupils; there are very few of us here who have not had some experience in this direction, and in the light of that experience, we do not see where we could have improved upon the selection, as well as upon our exactions as to the *régime* through which our pupils have had to pass during their preliminary training with us?

In many cases too little discrimination is painfully evident in the selection of the material for the coming dentist, this being supplemented by a rule of thumb pupilage, a superficial appreciation of fundamental principles is engendered which reflects unfavorably upon the early training of our prospective successors, and promotes a careless consideration of scientific subjects and data that goes far towards curtailing the value of many statements we hear made at our professional gatherings.

To quite an extent the remedy for this evil is in our own hands, did we but recognize the necessity for accepting as pupils only those who possess the natural qualifications for the study and practice of dentistry; and right here I would like to add that dentistry requires the greatest capacity for developing resource, and the ability to grasp the situation, and quickly apply correct principles, and make them subservient to the desired end in a more marked degree, than does any calling with which I am acquainted.

Our offices should not be made an asylum for any one who may be desirous of entering a profession simply because their parents or guardians are able to pay a premium and furnish the necessary funds to complete their prescribed collegiate career, for this in many instances proves the easiest way of finding them partial employment for a few years, and curtailing their opportunities for getting into mischief; it is this class that greatly contributes towards furnishing the dismal failures we so often see and hear of, and at the same time, by reflection, minimizes the well-meant efforts of our most earnest workers.

If the foregoing be true, then we must look at home for at least a partial remedy of this reproach, and, by a more judicious selection of pupils, weed out as far as possible this undesirable element of danger.

It seems to me, of late, too many of those to whom the young practitioner should look for inspiration and advice are astride that nondescript hobby which, to themselves, must have proved as great a farce as did a certain woolly specimen of the genus equina, and which has played the *rôle* of a misplaced switch in the lives of those who, having started out on a career of probable success and usefulness, and only lacking the years of experience which would have developed the positive element of their natures, thus resulting in outspoken, uncompromising champions of truth and the right, have hopelessly wrecked their prospects through the allurements of these mental acrobats.

In the medical profession we have the various schools represented, each one practising according to his belief in the tenets of his school, and accepted in the community in which he labors as a representative of said school. How different it is with our profession! looked upon in most communities as a calling composed of semi-professional mechanics, governed by but few well-defined lines of procedure, and whose success depends chiefly upon their ability to draw upon their natural resources, and when these are taxed to their utmost—as they not infrequently are—the results are only considered as coming from a "dentist."

The reason for this probably arises principally from two causes: First, our profession being practically in its infancy,—as compared with other branches of the healing art,—it can hardly lay claim to the time-honored dignity with which its compeer is favored; but if its subsequent progress is at all proportionate to its past history, it is destined to stand without a peer in the grand galaxy of science and art whose field is that of ameliorating human suffering. Secondly, the public have heretofore had such a peculiar practical experience with so-called dentistry, it is but reasonable to suppose they would look askance at any proposition that may now be made by our profession advancing its claims for recognition by them.

American dentists in Europe are continually having the status of American dental colleges hurled at them by the so-called professional literature of this side, and the value of their degrees questioned and ridiculed. American dental colleges like almost all other earthly institutions are open to improvement, and I have no doubt but their progressive superiority will be demonstrated in the future, as it has undoubtedly been in the past, continuing to furnish the pattern that is so poorly copied on this side, and which I am sorry to say at present is more of a cheap burlesque than a worthy replica; and while ridicule and poverty-stricken wit has taken the place of argument and logic in the European press, the fact remains that more bogus diplomas are held and more questionable means resorted to to obtain legitimate degrees by men on this side of the Atlantic than upon the other, and had it not been for the European market these diploma mills would have died a natural death.

As an item for the consideration of those whose cheap sneer is the only compliment they have at their disposal for the dental colleges of the United States, I would propound the following question: Why is it that the best and most progressive students, after completing their course on this side and having learned all their instructors were capable of imparting to them here, seek the halls of learning in America to secure that which they were unable to obtain here, where the best obtainable is supposed to be within easy reach?

And why is it, if American colleges are as superficial as they are said to be, their alumni hold the positions in the European capitals they do? I am constrained to believe we shall have to wait some time for a reply, the enforced silence of the press upon these points clearly endorsing public opinion as to the merits of the respective institutions and their representatives.

While enjoying the proud distinction and privilege of representing here the various institutions of dental education in America, we must exact of them the necessity of an advanced curriculum and technique, and the cultivation and promotion of a keener appreciation of professional dignity than that hitherto enjoyed by the average dental student; the building up of his professional career upon this basis will do much towards eliminating from our calling what has heretofore been a great element of weakness.

With all the restrictions surrounding the practice of dentistry in Europe, it is to America that the public on this side must look for the best exponents of our science and art, and, speaking more specifically of England, the supply hardly equals the demand, as there seems to be a decrease of active practitioners, and the number of registered pupils insufficient to fill deserted ranks.

While only having just touched upon some points where advance may be made as to our obligations in connection with the pupil question, and without suggesting an arbitrary remedy for the many evils that may exist in its present condition, and which will admit of much personal reflection and consideration, I will pass on to the brief consideration of the second proposition. The lack of stability in the ideas of young practitioners, I believe, is partly accounted for by some of the before-mentioned influences, supplemented by the off-hand utterances and vacillation of many of our older practitioners who frequently, to please some one else, blow both hot and cold, and make remarks that are allowed to pass unchallenged when they are known to be erroneous, or capable of very great modification, according to the existence or non-existence of certain circumstances.

If the recruits of our ranks were to indulge in the highly beneficial mental exercise of reasoning from cause to effect, and *vice versa*, a capacity for analysis would be promoted that would very materially assist them in readily detecting flatulent verbosity from well-evolved ideas, and assembling to their own advantage the essence of the subject under consideration, and discarding the almost impenetrable husk with which many good ideas are too frequently covered.

There are two studies that could be pursued to advantage by the average man, and would do much to wean him of the habit of making the random statements we so often hear; these are chemistry and metallurgy; and, strange to say, while these studies are embodied in all dental curriculi, how much time is devoted to the careful consideration of either? Both being dismissed in the most superficial manner; yet, being *exact* sciences, their importance from that stand-point alone would at once suggest the necessity of their thorough consideration.

This suggests to me the question, Where are the chemists of our profession? The reply is conspicuous by their absence; and while it remained for our profession to develop the possibilities of ether and nitrous oxide gas, it was not dentists who discovered either agent. The theorists and experimenters of our profession are rarely practical with their ideas, but very like statisticians looking for something, and, by a careful manipulation of methods or figures, find what they look for. And this not infrequently passes for scientific research, and correspondingly misleads any one whom a practical experience has not taught better. Hence the necessity of keeping an open and unbiased mind, one free and unprejudiced, weighing in the scale of private practice the problematical commodities so lavishly thrust upon us by men whose scientific capacity is often in the inverse ratio to their sublime self-assurance.

Let us, therefore, be guarded in our statements, and do not let us send forth as an axiom an idea that is based upon a single success, but let its value be proved by repeated exactions under different circumstances; let us be more accurate and thorough in the tabulation of cases; let us talk a little more of our failures, and trust our successes to take care of themselves, for some brother may be able to pilot us past the rock that stranded our initial effort, and help bring order out of chaos, and thereby assist us to round out our sphere of usefulness, and thus promote our highest ambition to be the greatest good to the greatest number.

